# Moving a Team-Based Freshmen Biomedical Engineering and Design Course Online

**DOI:** 10.1007/s43683-020-00005-0

**Published:** 2020-08-17

**Authors:** Eileen Haase

**Affiliations:** Department of Biomedical Engineering, Johns Hopkins University, Baltimore, MD 21218 USA

**Keywords:** Engineering, Undergraduate education, First year, Online teaching, Design, Team projects, COVID-19

## Abstract

Team projects and in-class interactions are the hallmark of a freshmen introductory course in biomedical engineering (BME). Our challenge was to continue team activities, mentoring, and the semester-long design project in a virtual environment after in-person classes ended due to the COVID-19 pandemic. This paper highlights some of the adaptations required to continue a large (*n* = 124) team-based course with students located throughout the world. Breaking up planned in-class activities into small individual assignments followed by team meetings gave groups a starting point for their discussions. Mentoring panels with upperclassmen were actually enhanced through the inclusion of alumni in a virtual environment. The end-of-semester anonymous survey results indicate that 81% of the freshmen agreed or strongly agreed that the course objectives were met, and 75% believed that the team assignments were useful learning experiences. In spite of the sudden shift to online learning, we were able to continue with both short-term and semester-long team activities.

**Electronic supplementary material:**

The online version of this article (10.1007/s43683-020-00005-0) contains supplementary material, which is available to authorized users.

## Challenge Statement

Engaging freshmen in team-based projects provides the personal, interpersonal, intellectual, and professional skills needed for a successful career.^3^ BME Basecamp is a one-credit team-based introduction to biomedical engineering, the design process, and many of the resources available to support our freshmen with their careers. The entire class meets once a week for 1-h, and teams meet on their own schedule outside of class. Teams brainstorm on new ways to design money, research potential solutions to health inequity challenges, and discuss case studies focused on ethical issues. The sudden move to online learning due to health and safety considerations caused by the COVID-19 pandemic required a change in the course syllabus, deliverables, and expectations, while keeping the initial course objectives.

We randomly placed 124 students in teams of five students on the first day of class. The class had a mix of in-class small group activities and long-term team assignments. The course objectives are listed in Table 1. The course emphasized developing the critical team work skills which are essential for a successful career.^1^ BME students learn most effectively from their peers and rely on classmates in each of their courses, especially design, to achieve success in the course objectives.^5^, ^12^, ^13^ Upperclassmen mentoring is another crucial component in guiding our BME students and starts the first day of class.^2^, ^7^ Team projects account for 70% of the course grading, including a semester-long healthy inequity design project. The design project is deliberately ill-defined to give students the flexibility to pursue an area passionate to them.^6^The remaining 30% of the grade is from individual assignments. *Our challenge was to continue team activities, mentoring, and the semester-long design project in a virtual environment after in-person classes ended*.

**Table 1:** Freshmen biomedical basecamp course objectives.

Students will be introduced to biomedical engineering through active introspective learning	
Students will learn to identify a desired need and define the biomedical engineering problem to be solved	
Students will work in teams. They will learn to develop team goals and to appreciate the complementary role and expertise of each team member	
Students will evaluate a hypothesis through experimental testing and statistical analysis	
Students will understand the guidelines for ethical and responsible use of human subjects and data for research	
Students will understand professional and ethical standards in the workplace	
Students will synthesize, summarize and explain technical content in written reports and oral presentations	
Students will recognize the need for self-assessment	

## Novel Initiative

After students returned home, there were some weeks we held only one large synchronous class during the regularly scheduled class time. The class was always recorded for students who could not attend. Other weeks we held numerous small group meetings at different times throughout the day to accommodate the students’ course schedules and time zones. Table 2 lists some of the course changes, while Online Appendix 1 provides the details of each of the team assignments. By necessity, a few activities were completely changed due to the COVID-19 pandemic, such as a scheduled field tip to Six Flags Amusement Park.

**Table 2:** Initial course plan and final plan after in-person meetings ended.

Topic	Initial plan	Final plan
Health Inequity Design Challenge	Needs Statement posted online Two minute elevator pitch on three health inequity issues and selection for project (no slides) Poster session on final design and initial prototype	Needs Statement posted online Elevator Pitches with Zoom in small groups (two slides) Seven minute PowerPoint presentation with Zoom in small groups Parents, family, and friends invited to Zoom presentations
Negative Feedback Control Loops	Interactive in-class lecture with small group activities on the Baroreceptor Reflex and control of blood pressure Class trip to Six Flags Amusement Park to observe negative feedback control of blood pressure (record heart rate and acceleration data)	Online lecture on negative feedback control systems. Teams discuss at least five human negative feedback control systems. Team analyzes one physiological system using negative feedback control loops.
Ethics	Individually, students complete online certifications on Human Subjects Research (HIPAA), Responsible Conduct of Research, Animal Care & Use, & Bloodborne pathogens In class lectures and case study discussions with lots of clicker questions and think-pair-share	Individually, students complete online certifications on Human Subjects Research (HIPAA), Responsible Conduct of Research, Animal Care & Use, & Bloodborne pathogens After the initial ethics lecture, students read selected NIH case studies [10], discussed as a team, and posted individual reflections.
Using Statistics in Medical Device Design	In class statistics lecture Teams design an experiment to obtain and analyze heart rate variability during a Six Flags trip.	Online statistics lecture Teams obtained Heart Rate Data at rest and during exercise (either with a smart phone app or with a pulse). Teams compared data within small groups (6-7 teams) using statistical analysis
Mentoring	Upperclassmen student panels on: – choosing a BME focus area – study abroad opportunities – undergraduate research – design teams	Online student panels (with alumni): – same sessions as originally planned – email addresses of panelists provided to freshmen – Q & A used the chat box in Zoom – sessions were recorded

A number of planned in-class activities were split into individual assignments, followed by a team assignment, modeled from the effectiveness of Team Based Learning.^9^ This helped students prepare for their team meetings and gave them a starting point during their limited time together. One example was the ethics module. Class discussions on case studies in ethics, which normally were held entirely during the one-hour class period, were split into an individual reading, team discussion, and then a written individual reflection.^10^ Table 3 lists the week-by-week deliverables for the semester-long health inequity design project. The transition to online occurred after week 6.

**Table 3:** Design project week-by-week deliverables.

Week	Deliverable: the design project was divided into a number of discrete steps; the transition to online occurred after week 6	Individual/team submission
1	Post *three* examples of health care inequity, provide references	Individual
2	Team of five students reviews all fifteen individual submissions and chooses their top *five* examples of health inequity	Team
3	Choose *one* of the five health inequity examples to focus on this semester and writes a “Needs Statement”, provide additional references	Team
4	Two minute elevator pitch. Seven teams present in an hour using Zoom.	Team
5	Peer review (rank self and teammates on project effort from 1 = best to 5 = no work)	Individual
6	Continue research on chosen project. Team decides who will research each area	Individual
	*Switch to online due to the COVID-19 pandemic*	
7	Develop table with design criteria (“must have” and “optional”)	Team
7	Mid-semester anonymous survey - identify student needs due to the pandemic	Individual
8	Delineate three potential solutions to needs statement	Team
9	Select top solution using design criteria	Team
10	Peer review (rank self and teammates on project effort from 1 = best to 5 = no work)	Individual
11	Post draft slides for final presentation	Team
12	Final Oral Presentation: Each of the five students on the team must present part of the 6-7 minute presentation. Seven teams present in an hour using Zoom.	Individual and team
13	Peer review (rank self and teammates on project effort from 1 = best to 5 = no work)	Individual
14	Anonymous survey (credit for completion)	Individual

Upperclassmen mentoring is an essential component of the course. Upperclassmen student panels discuss choosing a focus area, study abroad opportunities, gaining research skills, and design team projects. These panels provide freshmen a chance to ask questions and gain inside information about academic and career pathways. An unexpected advantage of holding these panels online is that they could now include alumni from across the country. There were three panels scheduled throughout the semester, two were in-person and one was virtual. Virtual mentoring is quite effective and also allows students anonymity when asking questions, which can encourage quiet students.^4^, ^14^

## Reflection

**Figure 1:**
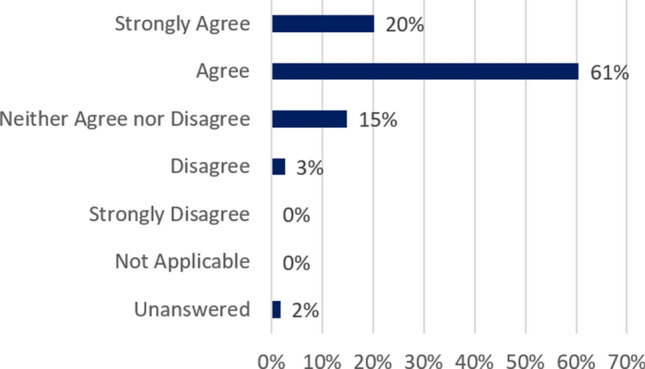
Based on an anonymous end-of-semester survey, 81% of students “agreed” or “strongly agreed” that the course objective listed in Table 1 had been met.

Our goal was to engage students in teamwork while solving vaguely-structured problems, a proven bonding and learning experience.^6^ Even with the change in plans, which included postponing the trip to Six Flags, 81% of students felt the course met its objectives (Fig. 1).

**Figure 2:**
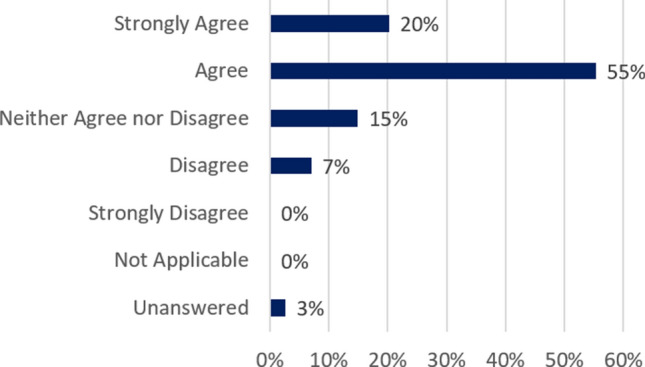
At the end of the course, 75% of students “agreed” or “strongly agreed” that the team assignments were useful learning experiences. Most of the course grade (70%) was based on team assignments.

The majority (75%) of students agreed that the team assignments were useful learning experiences (Fig. 2). In fact, students appreciated the effort to continue the team assignments. As one student noted, *Being with a team is something I took for granted while on campus.* There were three peer reviews during the semester to ensure that each member of the team was contributing to the assignment. The peer review is listed in Table 4. On a scale of 1 (best) to 5 (worst), the average peer review grade was between 1 and 2 for all three reviews. Students who received peer review grades of more than 3 by multiple team members were contacted by the TAs (first time) and faculty (second time).

**Table 4:** Peer review rating system.

For each of your teammates, including yourself, list their name and a number between 1 and 5 (i.e. Student NameA-1, Student NameB-2, Student NameC-1, Student NameD-2, Student NameE-1)	
(1) Always on time and prepared for team assignments, significantly contributes to discussions, does more than their share of the work	
(2) Almost always on time and prepared for team assignments, contributes to discussions, does their share of the work	
(3) Does not do *one* of the following: always on time and prepared for team assignments, significantly contribute to discussions, does their share of the work	
(4) Does not do *two* of the following: always on time and prepared for team assignments, significantly contribute to discussions, does their share of the work	
(5) Does not do *any* of the following: always on time and prepared for team assignments, significantly contribute to discussions, does their share of the work	

We required students to check-in each week during the scheduled course time(s). This proved to be a burden for some students, either due to the time zone, limited internet within their household, or the necessity to watch younger children, as noted in the quote below. We plan to give more flexibility in the future.

“*Don’t take mandatory attendance even for USA students. Some students have spotty internet connections or may have other conflicts such as taking care of family members or household duties. Besides that, this class functions pretty well online*.”

A number of students suggested that teams should be formed based on time zones since we had quite a few students in Asia. However as one astute student noted:

“*It would be really helpful for teams to possibly be broken up by time zone since it would be easier for them to collaborate and/or communicate. I understand, however, that this may possibly lead to a lack of diversity in terms of background for the teams*.”

While it is tempting to form teams based on convenience, freshmen would miss the chance to work with classmates from around the world. We have decided to keep random teams for fall 2020.

The teams presented their design ideas synchronously using Zoom in an Elevator Pitch and a final PowerPoint presentation. The entire team of students were required to show their faces during graded presentations. While these went well enough, students would have preferred to give their talks in person.

“*The only bummer was … the pitch presentation not being in person - it’s definitely easier but would definitely learn more by giving pitches in person.*”

Parents and friends were invited to the Zoom presentations. This was the policy for final presentations in a number of our BME courses, and an advantage of online presentations that many students, faculty, and parents enjoyed. However, the freshmen did not take advantage of this option. Future online offerings could have a Livestream so that parents can join remotely without appearing on Zoom, which might have made some students uncomfortable.

The teams did not have the opportunity to actually “prototype and test” their design projects, which are listed in Table 5. This was especially frustrating since the BME program is focused on research and design, and many of our students join design teams as freshmen. Future online offerings of the class will mail an Arduino Student Kit home to the freshmen. There will be an individual design project using a temperature sensor, followed by a team design project of their choice. These kits will provide a hands-on design experience and allow teams to work on projects synchronously. Adding a hands-on component in an online design course is an important element for student satisfaction.^8^

**Table 5:** List of team health inequity projects.

Definition of Health Inequity from World Health Organization: Health inequities are differences in health status or in the distribution of health resources between different population groups, arising from the social conditions in which people are born, grow, live, work and age. Health inequities are unfair and could be reduced by the right mix of government policies.^15^	
Health Inequity Challenges based in the US	Health Inequity Challenges based in LMIC (Low and Middle Income Countries)
Opioid abuse	Child healthcare
Prenatal healthcare	Infant mortality/low birth weight babies
Access to healthy and fresh foods	Needle sterilization
Maternal mortality rates	Water quality
Overcrowded living conditions	Water borne diseases
HIV infections among African Americans	Tuberculosis diagnosis and treatment
EMT shortages	Malaria
Shortage of doctors in rural areas	
Mental health illness diagnosis and suicide rates	
Healthcare access for Aboriginal populations	
Veterans mental health resources	
Sex education programs	
Accommodations for differently-abled patients	
Transportation options for patients with disabilities	*Note: some teams had similar projects*

This one-credit spring course will be offered next fall (2020) as a two-credit online course, which will hopefully allow some in-person design work. We have arranged for thirty upperclassmen to serve as lab managers next fall, at least one per team of five freshmen, to provide one-on-one guidance for each of the assignments. The lab managers will not be responsible for any of the grading, only mentoring. This arrangement benefits both the upperclassmen mentors, who gain valuable teaching and leadership experience, and the freshmen, who receive low-key advice.^7^, ^11^

## Conclusion

In spite of the sudden shift to online learning, we were able to continue with both short-term and semester-long team activities. The mentoring panels with upperclassmen were enhanced through the inclusion of alumni in a virtual environment. Breaking up planned in-class activities into small individual assignments followed by team meetings gave groups a starting point for their discussions. Peer reviews ensured that team members were held accountable for their share of the projects. In our next offering of this course, we plan to increase the number of upperclassmen mentors to at least one per team of five freshmen and mail the students Arduino kits to work on a design project at home. The transition to online still allowed students to meet the course learning objectives and, most importantly, gain team work skills.

## Electronic supplementary material

Below is the link to the electronic supplementary material.

Supplementary material 1 (PDF 1938 kb)
