# Supplementary material for: Moving a Team-Based Freshmen Biomedical Engineering and Design Course Online
Source: Biomed Eng Educ. 2020 Aug 17;1(1):31–6. doi: 10.1007/s43683-020-00005-0 (PMC7430930; doi:10.1007/s43683-020-00005-0)
Supplement: Supplementary file 1 — Supplementary material 1 (PDF 1938 kb) [file 43683_2020_5_MOESM1_ESM.pdf]

Appendix 1: Assignments and Rubrics

This appendix includes the assignments, and in many cases the rubrics used to grade the assignments. There are five assignments:

I. Health Inequity Design Project Elevator Pitch
II. Heath Inequity Design Project Final Presentation
III. Case Studies on Ethics
IV. Negative Feedback Control Systems
V. Statistical Analysis of Heart Rate Data

I. Design Project Elevator Pitch: How will you solve a problem in health inequity?

1. Sign a time slot for your team at: Elevator Pitch - Doodle poll sign-up (note: only one person from the team should sign up for a time, and teams can only sign up for one time slot). If there is not a time that everyone can attend then sign for a time that most team members can attend.
2. You have 1.5 to 2 minutes (90 to 120 seconds). Running over or under that amount of time will result in points off. For such a short presentation, it is best to have just one person speak. Normally an elevator pitch is done without slides or uses a quick sketch on a whiteboard. Normally an elevator pitch is less than a minute, but in general, the shorter the pitch, the harder it is to get it right. In the interest of time and efficiency, you can use slides (not required). For such a short time, you should have **no more** than four slides. The first one doesn't really count: it is your team number and names. Everyone should have this.

   The next three slides **might** be divided as follows (you can choose to have only one slide with your names if you prefer - these are only suggestions):

   - What is the problem you are addressing? Why is it a problem? How many people are impacted? What is your "needs statement" (40-60 seconds)
   - What are some ways you might address this problem? You should list your three favorite ideas - don't get into details (20-30 seconds)
   - Which idea do you think is best and why (one or two details)? (30 seconds)
3. Practice, practice, practice your pitch. The spokesperson for the group should have a script and the team members should provide feedback during **many** practice sessions. This is an important project that you have chosen to research – health inequity. The audience should feel empathy for whoever is impacted (this is where you might have a powerful slide illustrating the health inequity). At this point you have no idea whether your solution can possibly work. The point with an elevator pitch is to get excitement for your idea so that it can get some initial funding. Listing three choices and choosing one shows that you have given this some thought, not that you have solved the problem.
4. Rubric: out of ten points you will be graded on five items; keeping time, generating empathy, defining a problem (needs statement), listing three potential solutions, choosing one solution (2 points possible for each component, 5 x 2 = 10 points total):
   - 2 points = well defined
   - 1 point = good, but ran over time, missing something, confusing presentation
   - 0 = not addressed

<u>II. Design Project Final Presentation:</u> How will you solve a problem in health inequity?

1. Sign a time slot for your team at: Final Presentation - Doodle Poll sign-up (note: only one person from the team should sign up for a time, and teams can only sign up for one time slot). You should plan to participate for the entire hour. There will be four sessions with seven teams each. The times are Thursday, April 30, from 7:00-8:00 PM, and Friday, May 1, from 10:30-11:30, 11:30-12:30, and 3:00-4:00 PM. I tried to cover a range of time zones.
2. You have **6-7 minutes**. Running over or under that amount of time will result in points off. You must have **every member** of the team speak at least once. PRACTICE to ensure that you do not go over or under the time limit.
3. Your opening slide should have your team number, team member names, and project title.
4. Your slides should build from simplicity to complexity slowly (remember the example from the lecture on giving oral presentations).
5. You will be building on the slides from your elevator pitch, but since you have more time you can get into more detail:
   - What is the problem you are addressing? Why is it a problem? How many people are impacted? What is your "needs statement"? Some of these slides can be identical to your elevator pitch.
   - What are some ways you might address this problem? You came up with three ideas for your elevator pitch. Please list these (you can list more than three) on a chart and the criteria used to decide to focus on your final solution. Note: You do not need to keep the same solution from your elevator pitch, although you should indicate what additional information led you to change from your original solution.

   A sample summary chart is shown below:

**Comparison of solutions to Problem X**			
**Solution**	**Cost**	**Time to implement**	**Training required**
Idea 1	$$$ (expensive)	Very Slow (weeks)	Requires clinician
Idea 2	$	Slow (days)	Trained community health worker (volunteer)
Idea 3	$	Fast (minutes)	Trained community health worker (volunteer)

6. You have more time in this talk to spend on your final solution. Why is this one the best (you can start with the results from your chart)? There are a number of criteria which are important in deciding on a final design solution. These are usually divided into "Must have features" and "desirable features". Some typical design criteria are listed here, but you can certainly add more of your own:
   i. Accessibility (can the people who need this solution get it?)
   ii. Safety (is this solution safer than the current solutions? Can anyone get hurt with this solution?)
   iii. Cost (is it affordable? Could this be covered by insurance or NGO donation?)
   iv. Cultural Sensitivity (will the people who need this solution adapt it?)
   v. Robustness (does everyone who uses the solution get the same results?)
   vi. Resources (what is required to implement this solution – a building, website, hardware?)
   vii. Time (how long will it take to implement this idea? How long for someone to get a result?)
   viii. Skill required (does this solution need a trained caregiver? A laminated instruction card?)
   ix. Low power (can run off a cell phone battery)
   x. Your own criteria….
7. Practice, practice, practice your talk. Each spokesperson on the team should have a script and practice on their own and with the group. You don't want to waste time transitioning from one speaker to another. Practice during **<u>many</u>** practice sessions while timing yourselves. This is an important project that you have chosen to research – health inequity. The audience should feel empathy for whoever is impacted (this is where you might have a powerful slide illustrating the health inequity). At this point you still have no idea whether your solution can possibly work. The point with this presentation is to get excitement for your idea so that it can get some initial funding. Listing three choices and choosing one shows that you have given this some thought, not that you have solved the problem.

<u>II. Design Project Final Presentation:</u> How will you solve a problem in health inequity? - continued

8. Rubric: out of twenty points you will be graded on ten items;
    i. keeping time
    ii. each team member speaks effectively (has clearly practiced)
    iii. generating empathy
    iv. defining a problem (needs statement)
    v. listing at least three potential solutions in a chart
    vi. defining your design criteria
    vii. choosing one solution
    viii. building your slides (simple to complex)
    ix. using active voice
    x. telling the right story (know your audience – freshmen in BME)

   Two points possible for each component, 10 x 2 = 20 points total:
    - 2 points = well defined,
    - 1 point = good, but ran over time, missing something, confusing presentation,
    - 0 = not addressed
9. Post your slides and your script on Blackboard (your script can be in the notes section of your PowerPoint slides)
10. You may share the Zoom link with your family and friends

III. Case Studies on Ethics: **How do you decide if a solution is ethical?**

Read the case studies assigned to your group before you meet. Divide up as a team with people taking different sides of the issue. You may look for references, if appropriate, to support your position. Prior to reading and discussing your papers, read this file, which summarizes material from NIH.

a) Teams should **select** one article from the AMA Journal of Ethics, January 2020 on "Culture, Context, and Epidemic Containment" to discuss as a team.
b) Each team should **select** one additional article from the list of "optional topics". In discussing these articles, split your team into two and "choose a side". I suggest each student on the team picks one article to read, discussing it online with their team, and then choose one to focus on as a group.
c) Each student should write a reflection on **both** case studies. Your reflection should include both perspectives and answer some of the questions and points below.

How Should Cases be Analyzed?

Many of the skills necessary to analyze case studies can become tools for responding to real world problems. Cases, like the real world, contain uncertainties and ambiguities. Readers are encouraged to identify key issues, make assumptions as needed, and articulate options for resolution. In addition to the specific questions accompanying each case, readers should consider the following questions:

1. Who are the affected parties (individuals, institutions, a field, society) in this situation?
2. What interest(s) (material, financial, ethical, other) does each party have in the situation? Which interests are in conflict?
3. Were the actions taken by each of the affected parties acceptable (ethical, legal, moral, or common sense)? If not, are there circumstances under which those actions would have been acceptable? Who should impose what sanction(s)?
4. What other courses of action are open to each of the affected parties? What is the likely outcome of each course of action?
5. For each party involved, what course of action would you take, and why?
6. What actions could have been taken to avoid the conflict?

Is There a Right Answer?

Acceptable Solutions
Most problems will have several acceptable solutions or answers, but it will not always be the case that a perfect solution can be found. At times, even the best solution will still have some unsatisfactory consequences.

Unacceptable Solutions
While more than one acceptable solution may be possible, not all solutions are acceptable. For example, obvious violations of specific rules and regulations or of generally accepted standards of conduct would typically be unacceptable. However, it is also plausible that blind adherence to accepted rules or standards would sometimes be an unacceptable course of action.

Ethical Decision-Making
It should be noted that ethical decision-making is a process rather than a specific correct answer. In this sense, unethical behavior is defined by a failure to engage in the process of ethical decision-making. It is always unacceptable to have made no reasonable attempt to define a consistent and defensible basis for conduct.

References:
https://oir.nih.gov/sourcebook/ethical-conduct/responsible-conduct-research-training/annual-review-ethics-case-studies/
https://journalofethics.ama-assn.org/cases

<u>IV. Negative Feedback Control Systems:</u> How humans maintain short term control of Blood Pressure

1. Watch the Panopto recording on Negative Feedback Control of Blood Pressure.
2. Blood pressure is just one variable that is controlled using negative feedback. Brainstorm with your team. What are at least **<u>five</u>** different variables in your body that are controlled?
3. With your team, choose your favorite controlled variable and do a little research. Sketch a negative feedback control loop and for your system identify each of the following:
    - Measured variable (i.e. blood pressure)
    - Set point (for blood pressure it was 100 mmHg)
    - System (for blood pressure it was the cardiovascular system: heart, arterial vessels, venous vessels)
    - Feedback mechanism (i.e. blood pressure is measured within the body at the carotid and aortic baroreceptors)
    - Disturbances (which might cause the measured variable to change)

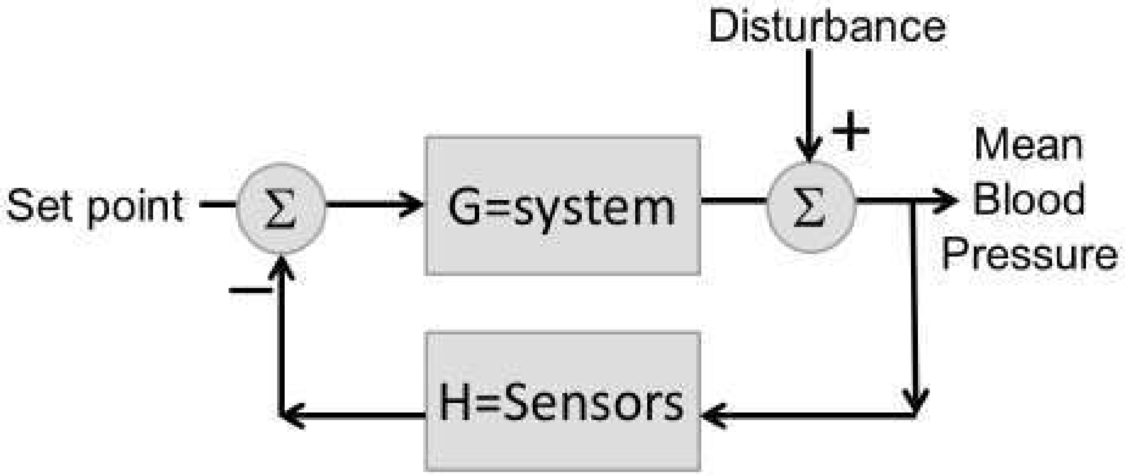

Figure 1. Negative Feedback Control System Loop

<u>V. Statistical Analysis of Heart Rate Data</u>: Difference between rest and exercise

1. Take at least ten resting heart rate measurements (one a minute for ten minutes if using an app, or ten fifteen second counts using your pulse).
2. Take at least ten exercising heart rate measurements. Heart rate should to increase to over 120 beats per minutes, or double your resting heart rate, whichever is less.
3. Find your average HR at rest and during exercise (mean $\pm$ st dev) and calculate the difference (average HR during exercise – average HR during rest).
4. Pool data with the rest of your team. What is the team's average HR at rest, exercise? What is the average difference? Is there any significance to the difference? What type of statistical test should you use?
5. Pool data with the other teams in your cohort. Answer the questions from above with your cohort (What is the cohort's average HR at rest, exercise? What is the average difference? Is there any significance to the difference? What type of statistical test should you use?)

Apps to measure heart rate using your smart phone:
Cardiio iOS app, Instant Heart Rate Android app, Cardiograph iOS app
https://www.cnet.com/how-to/how-to-track-your-heart-rate-with-a-smartphone/

Don't want to use an app? Then take your pulse by hand:
https://www.webmd.com/heart-disease/heart-failure/watching-rate-monitor#1
